# Negative linear compressibility of a one-dimensional interpenetrated metal–organic framework

**DOI:** 10.1039/d6sc02574a

**Published:** 2026-07-08

**Authors:** Sarah L. Griffin, Alexandra Longcake, Mateusz Mojsak, Adam A. L. Michalchuk, Michael R. Probert, Neil R. Champness

**Affiliations:** a School of Chemistry, University of Birmingham Edgbaston Birmingham B15 2TT UK n.champness@bham.ac.uk; b Chemistry, School of Natural and Environmental Sciences, Newcastle University Newcastle Upon Tyne NE1 7RU UK

## Abstract

A metal–organic framework (MOF), UoB-200, with a necklace topology that exhibits rare 1D → 1D parallel interpenetration is reported. UoB-200 exhibits significant negative linear compressibility (NLC) over a pressure range up to 3.44 GPa. When pressure is applied, elongation along the crystallographic *c* axis is observed *via* a wine-rack mechanism. The experimental results are modelled by simulations, which closely reproduce the mechanical response and suggest that the presence of solvent within the UoB-200 pores has minimal impact on the NLC behaviour at comparatively low pressures.

## Abstract 2

The MOF UoB-200 features an interpenetrated necklace topology and exhibits significant negative linear compressibility under pressure *via* a wine-rack mechanism, representing a new class of one-dimensional materials with such mechanical behaviour.

## Graphical abstract

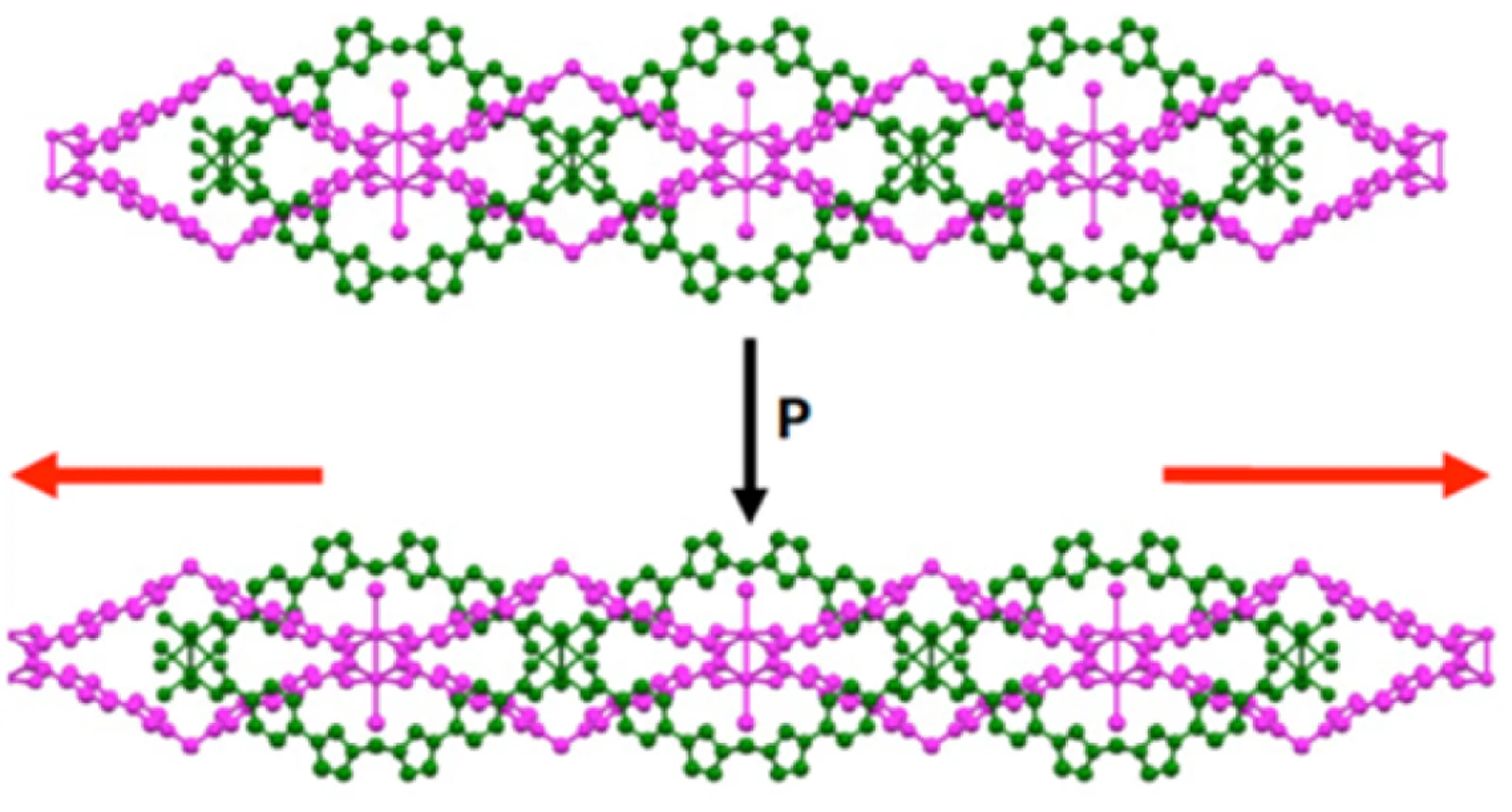

## Introduction

Reticular chemistry offers the potential to synthesise new materials with controllable structure. The successful implementation of the reticular approach has been demonstrated for a variety of materials, but particularly for metal–organic frameworks (MOFs).^1–4^ The breadth of properties displayed by MOFs is remarkable and arises from the ability to design new frameworks with distinct topologies and structural features.^5–8^ A notable feature of MOFs is the ability of some frameworks to exhibit flexibility and breathing^9,10^ and unusual behaviour under pressure.^11,12^ Such properties have received significant attention over recent years and examples of behaviour such as negative linear compressibility (NLC)^13^ has been observed for a small number of MOFs.

NLC is demonstrated by materials that expand in one or more directions whilst compressed under uniform, hydrostatic, pressure.^12^ Such behaviour has been observed for a range of materials, from simple molecular systems^14–17^ through to inorganic solids, such as metal oxides^18–21^ and halides,^22–25^ and metal cyanides.^26–29^ MOFs offer an attractive target for the preparation of materials which exhibit NLC behaviour and examples of such systems have been reported.^11–13,30–36^ A significant proportion of the MOFs that exhibit NLC exploit a wine-rack mechanism that accommodates the extrinsic pressure on the framework.^11,12,31–34^ The flexibility of the MOF, through the wine-rack motion, facilitates the additional energetic imposition without leading to amorphization of the MOF.^11,37^ A common feature of the MOFs that exhibit wine-rack flexibility are square, or loop, shaped subunits that distort through flexibility of the metal-based corners.^11,12,31–34^ The frameworks that exhibit NLC behaviour are three-dimensional in structure and thus we decided to explore whether a MOF with lower dimensionality, such as one-dimensional (1D) MOFs, would exhibit wine-rack behaviour under high pressure.

**Fig. 1:**
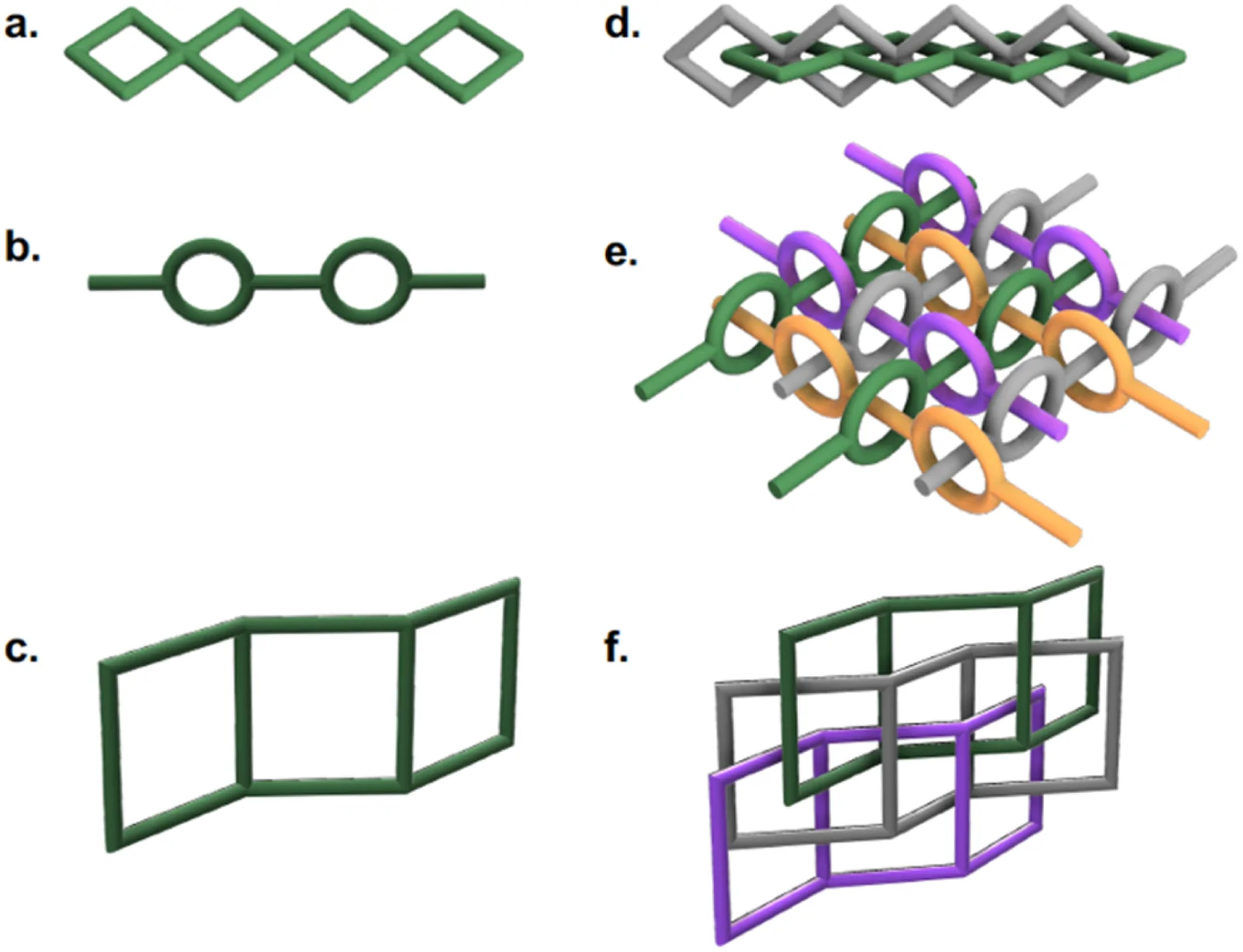
1D MOFs and potential interlocked structures. (a) Necklace structure with loops joined at a single point, as observed for UoB-200; (b) necklace structure with loops joined through an additional linker; (c) ladder MOF structure; (d) parallel interpenetrated 1D necklaces, as observed for UoB-200; (e) perpendicular polycatenation of necklaces with loops joined by an additional linker; (f) parallel polycatenation 1D ladders.

Thus, we sought to explore 1D MOFs that contain loop subunits, such as necklaces (Fig. 1a and b) or ladders (Fig. 1c). Due to the presence of closed-loops in such MOFs, entanglement can be observed in 1D systems, with the loops hosting other loops or chains. Ladder-based MOFs (Fig. 1c) were amongst the first systems to be studied in detail and examples of both polycatenation and interpenetration,^38–47^ and even examples of self-catenation (or knotting),^48^ have been reported. See ref. 38 and 39 for a discussion of the terminology of polycatenation and interpenetration. Either polycatenation or interpenetration of ladders is observed in both parallel interpenetration (1D → 1D),^46^ parallel polycatenation (1D → 2D)^47^ (Fig. 1f)) and perpendicular polycatenation (1D → 3D^41–45^) arrangements leading to distinct combined structures.

MOF necklaces fall into two main categories, those in which the loops are joined at a single point (Fig. 1a) and those which are cross-linked through an additional linker^49^ (Fig. 1b). Polycatenation or interpenetration of necklaces can take place in a similar fashion to ladders, parallel interpenetration (1D → 1D) (Fig. 1d) and perpendicular polycatenation (1D → 2D/3D) (Fig. 1e). It was proposed in 2001 that (1D → 1D) interpenetrated MOFs could be formed from necklaces or ladders (Fig. 1d and f respectively).^50^ Despite the prediction of such interpenetrated arrangements these systems remain rarely observed.^46,51^ Indeed, a necklace that exhibits parallel interpenetration (Fig. 1d), has been reported only on one previous occasion.^13^ For one of the most fundamental interpenetrated structures, proposed over 20 years ago, it is surprising that the parallel interpenetrated necklace structure is so rarely reported. Herein, we report the synthesis and characterisation of a one-dimensional necklace with (1D → 1D) parallel interpenetration and explore the behaviour of this system as a function of pressure and temperature.

## Results and discussion

**Fig. 2:**
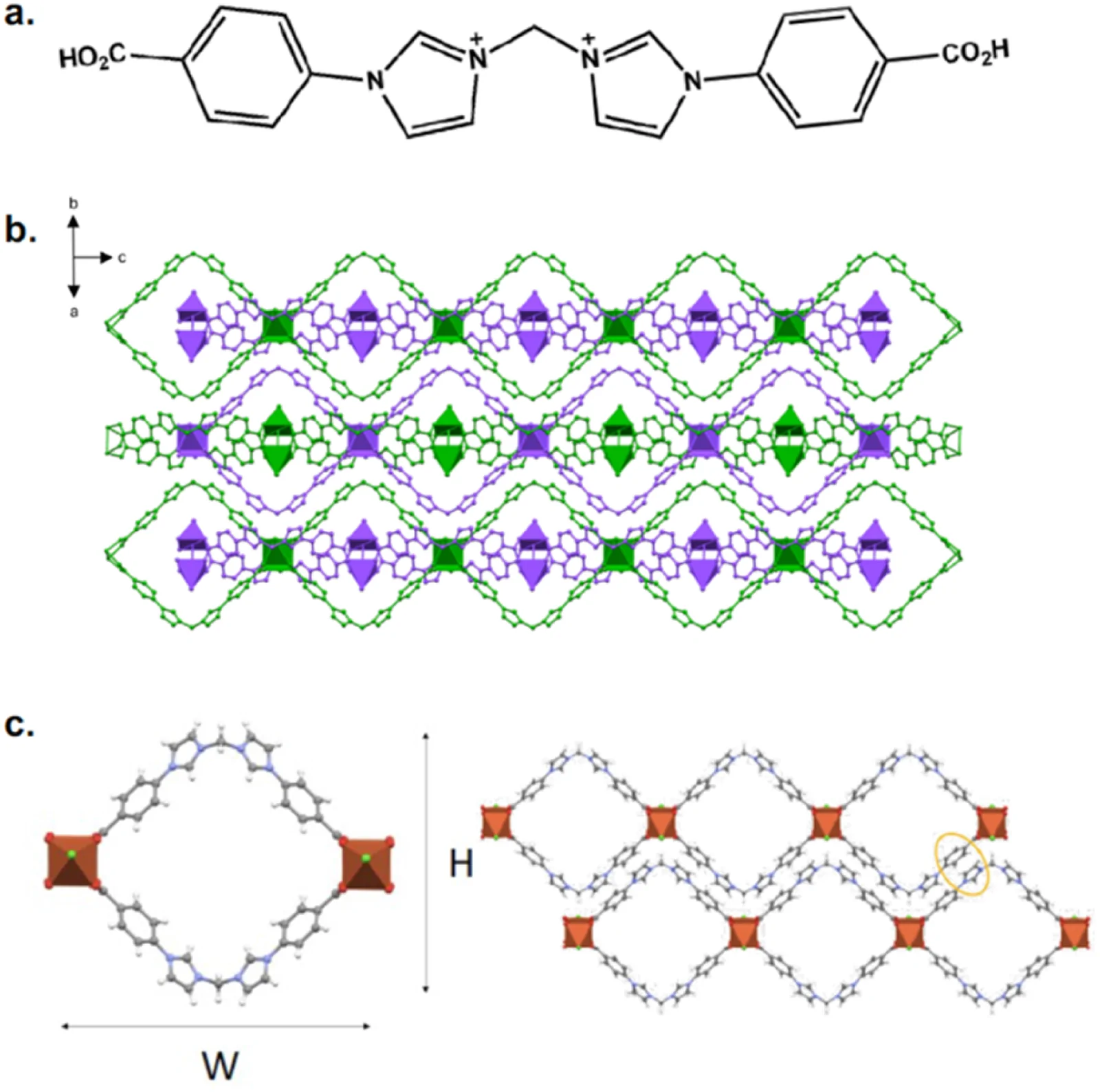
The structure of UOB-200. (a) The organic linker L used in the synthesis of UoB-200. (b) The crystal structure of UoB-200 as viewed down the [110] direction, with the symmetry related interpenetrated layers A and B depicted in green and purple, respectively. (c) A depiction of a single linked unit of UoB-200 with the aspect ratio of the pore defined as *W* : *H* (width : height), beside a single layer of UoB-200 as viewed down the [110] direction, with the π–π interactions discussed circled in orange.

The MOF, UoB-200, was crystallised *via* the layering of an ethanol solution of Cu(NO_3_)_2_ and an aqueous solution of the chloride salt of the linker L, (L)Cl_2_ (Fig. 2a). Over the course of one week, both blue plate and green block crystals formed. Single crystal X-ray diffraction (SCXRD) data was collected for the blue-plate crystals, showing them to be a 1D chain structure reported by Kong *et al.*^52^ SCXRD data collected for the green block crystals showed the formation of a new interpenetrated necklace MOF, UoB-200 (Fig. 2b and c). Crystallising in the space group *I*4/*m*, the structure comprises of bimetallic Cu(ii) paddlewheels coordinated by four linkers and chloride ions occupying the axial sites, leading to 1D chains of coordinated rings, or a necklace (Fig. 1a). Upon viewing the packed crystal structure, it becomes apparent that each 1D chain is arranged such that it allows the threading of a second chain, leading to two independent chains interlocking in an interpenetrated fashion, *i.e.* parallel (1D → 1D) interpenetrated necklaces (Fig. 1d). Charge balance is maintained by additional, uncoordinated, chloride anions positioned within the pockets of the structure.

**Fig. 3:**
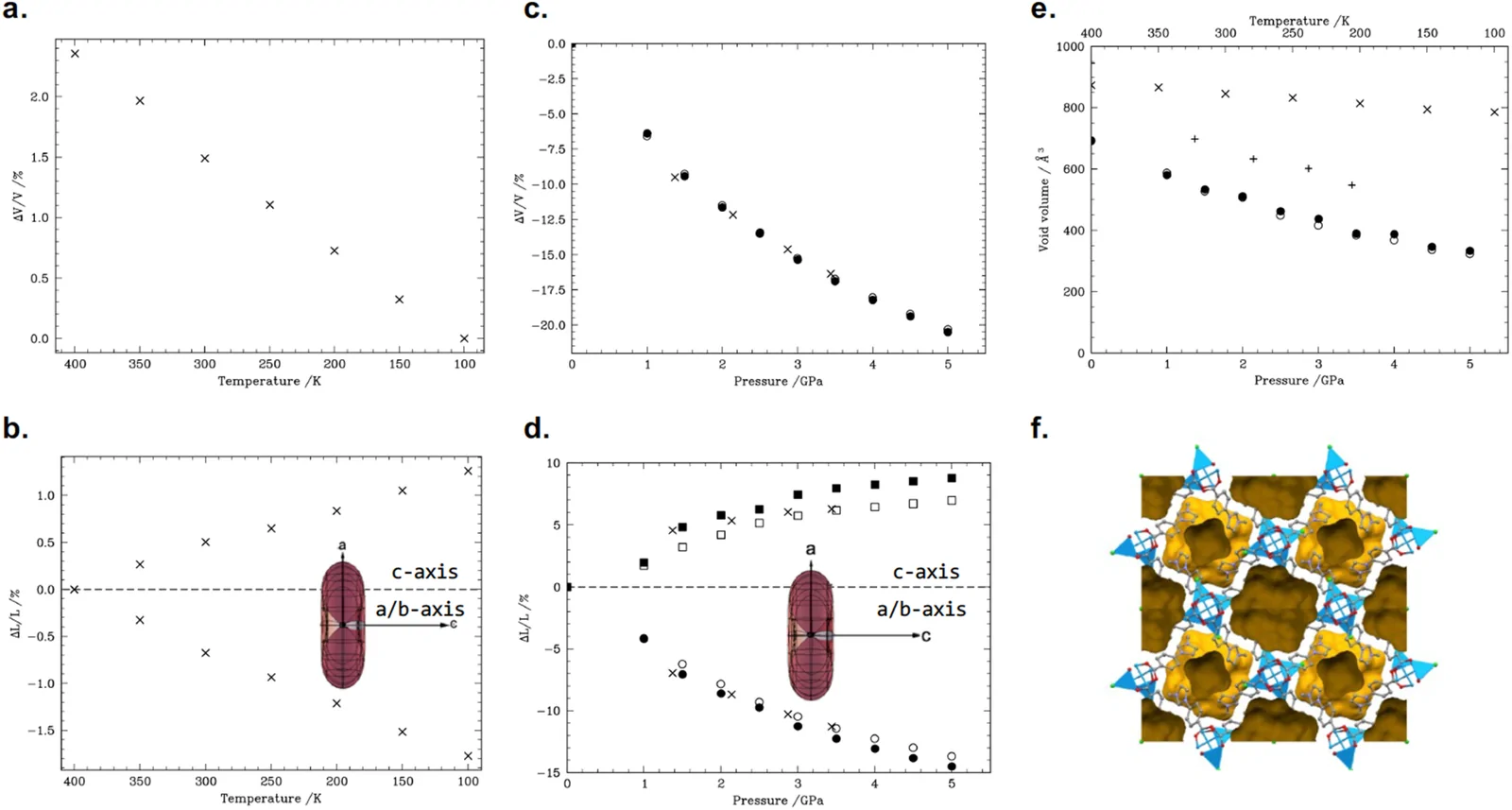
The effect of stress on the structure of the conventional unit cell of UoB-200. (a) The effect of temperature on the unit cell volume; and (b) on the crystallographic axes. The inset shows the thermal expansion indicatrix^50^ along the crystallographic *b* axis. (c) The effect of hydrostatic pressure on the unit cell volume; and (d) on the unit cell axes. The inset shows the compressibility indicatrix along the crystallographic *b* axis. (e) The effects of temperature and pressure on the void volume, indicated as yellow in the associated crystal structure diagram (f). Experimental variable temperature data are shown as grey crossed circles. In all plots, simulation data are given as PBE-D3 (closed symbol) and PBE-D4 (open symbol). Experimental data are given as crosses (in *e*, variable temperature data as *x* and variable pressure as +).

UoB-200 exhibits a structure that resembles a sub-component of a wine-rack structure,^11,12,31–34^ and so we investigated how the structure behaved upon variation in temperature and pressure (Fig. 3). Upon desolvation of a UoB-200 single crystal, achieved by holding a single crystal at 400 K for approximately 6 hours, the unit cell volume decreased from 4784.38(13) Å^3^ to 4650.56(16) Å^3^, a reduction of 2.8%. However, desolvation did not lead to a change in the overall crystal packing arrangement. Upon cooling the desolvated crystal from 400 K to 100 K, the crystallographic *a* axis of UoB-200 exhibited a reduction from 16.4259(3) Å to 16.1326(2) Å (1.7% decrease), whereas the *c* axis increased from 17.6424(7) Å to 17.8644(4) Å (1.3% increase), indicating a wine-rack mechanism of contraction. Following the desolvation step, SCXRD data were collected across a range of temperatures to assess the structure's flexibility. The desolvated sample was cooled to 100 K and then data was collected in 50 K increments up to 400 K, and then in 100 K steps upon cooling back to 100 K (Fig. 3a and b). From 100 K to 400 K, the unit cell volume increases by *ca* 2.4% from 4650.56(16) Å^3^ to 4760.1(3) Å^3^.

As a comparison to the effects of cooling, we subsequently compressed a single crystal of UoB-200 under hydrostatic conditions in a diamond anvil cell, to a maximum pressure of ∼3.44 GPa (Fig. 3c and d), see SI for details of experimental approach. Over this pressure range, UoB-200 compressed monotonically, with the unit cell volume decreasing by 16.3% overall. This unit cell volume change was accompanied by a decrease in the *a* = *b* crystallographic axes by 11.3%, and an expansion of the crystallographic *c* axis by 6.3%. Thus, UoB-200 exhibits NLC behaviour in the form of uniaxial negative compressibility, in common with 3D wine rack-like geometries.^11,12,31–34^ Above 4 GPa UoB-200 underwent amorphization, a process which is common for MOFs at the limit of their mechanical stability.^53–55^

To explore the effects of pressure on the UoB-200 geometry, we examined how the shape of the pores in UoB-200 are distorted by pressure. Each of the symmetry equivalent chains exhibit the ‘wine rack closing’ behaviour, whereby the height of the pore contracts as the width expands. To quantify this distortion in the pore shape, the ‘aspect ratio’ of the pore width (*W*) and height (*H*) was inspected (Fig. 2c). The height of the pore is defined in this case as the distance between the two apical carbon atoms adjacent to the imidazole rings, and the width as the distance between the centroids of the two copper atoms within the copper paddlewheel cores, respectively.

Under ambient conditions, the *W* : *H* aspect ratio was calculated as 1.18 : 1, increasing significantly by 3.44 GPa to 1.47 : 1, indicating a significantly distorted pore shape. The distortion of the pore is enabled primarily by a variation in the bond angles at the paddlewheel corners of each loop (*cis* O1–Cu1–O2 bond angles contract from 88.07(9)° (ambient) to 82.6(7)° (3.44 GPa)) whilst comparatively small variation is seen in the angle at the apex of the organic linker (N2–C11–N2 bond angle = 111.4(4)° (ambient); 108(3)° (3.44 GPa)). Statistically significant coordination sphere distortions around metal centres are commonly observed when coordination complexes are subjected to pressure, due to the more diffuse and flexible natures of these bonds.^56,57^ The aromatic π–π interactions between 1D necklaces (Fig. 2c) also exhibit a slight compression upon the application of pressure, with the vertical separations between the planes of offset benzene and imidazole rings of adjacent organic linkers decreasing from 3.87(2) Å under ambient pressure to 3.48(3) Å at 3.44 GPa, as is typical for such face-to-face π–π interactions in response to pressure.

**Fig. 4:**
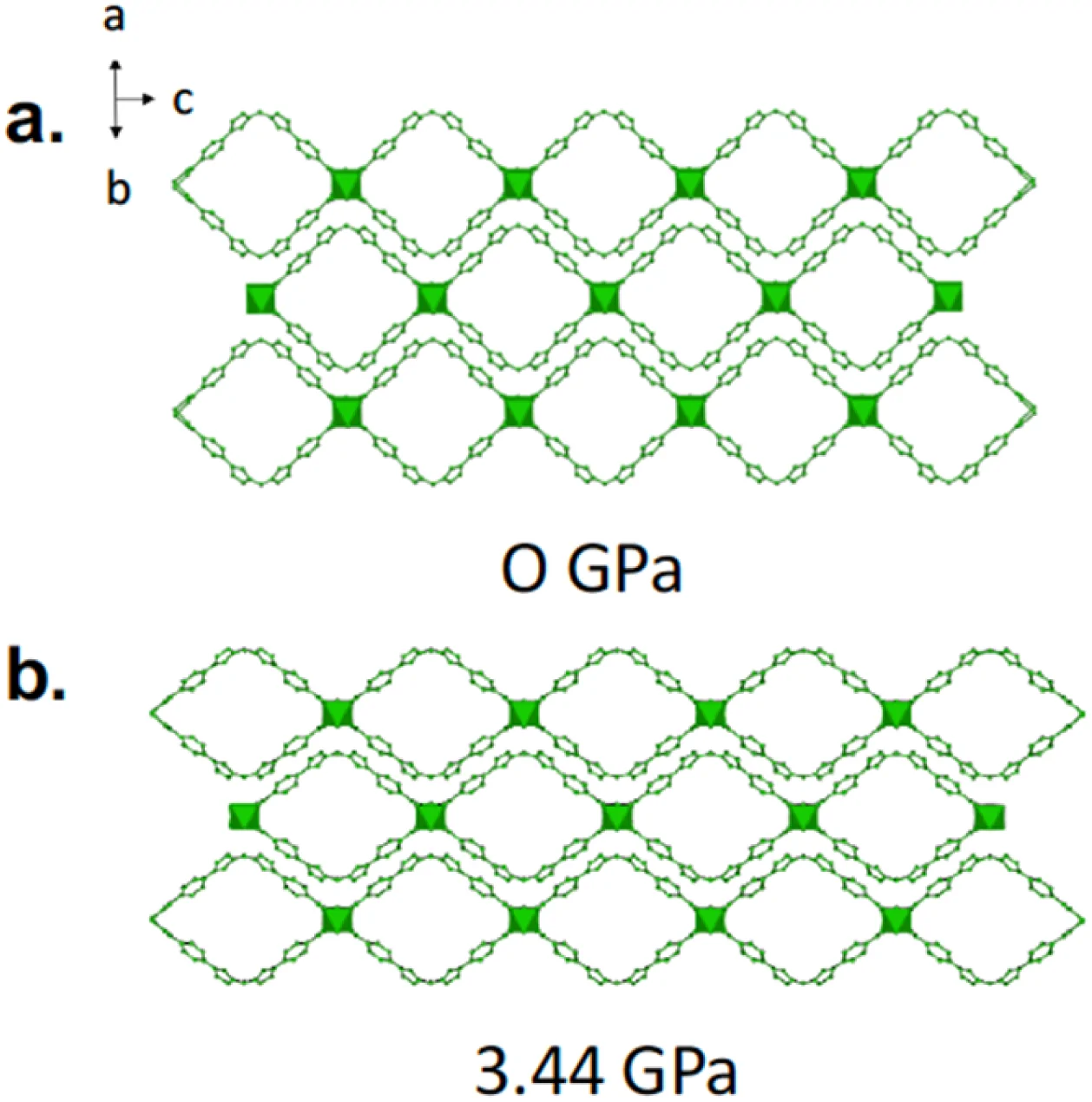
The structure of UoB-200 as viewed down the [110] direction, showing the ‘wine rack’ compression exhibited between (a) 0 GPa and (b) 3.44 GPa (only one symmetry equivalent layer is shown, for clarity).

Two alternating pore sizes are present in UoB-200, visible when the structure is viewed down the *b* axis (Fig. 4a), sitting within the (Cu_2_)_2_(L)_2_ necklace loop and between adjacent chains, located between the paddlewheel of one chain and the ligand of the other. At ambient pressure, the larger and smaller solvent accessible void spaces were calculated to be approximately 816 Å^3^ and 175 Å^3^, respectively.^58^ These pores presumably contain a mixture of ethanol and water (due to the crystallisation mother liquor), but the exact contents within the pores could not be resolved crystallographically because the solvent was highly disordered, even at high pressures. The number of electrons recovered from the solvent accessible void space of UoB-200 across the sampled pressure range was consistent, suggesting that as the structure was compressed, the solvent was not forced out of the voids. As the structure was compressed, the total void volume of UoB-200 decreased from approximately 991 Å^3^ at ambient pressure to 544 Å^3^ at 3.44 GPa (Fig. 4b).

To extend our study of the high-pressure behaviour of this new coordination polymer topology beyond the experimental limitation of *p* ≈ 3.5 GPa, we explored the high-pressure behaviour of UoB-200 using density functional theory (DFT) simulation at PBE-D3 and PBE-D4 levels of theory. Our DFT geometries underestimate the low-temperature (100 K) experimental unit cell volume of de-solvated UoB-200 (experimental primitive unit cell 2325.28 Å^3^) by only 0.3% for PBE-D3 and 1.7% for PBE-D4. This indicates that our simulation models provide a good representation for UoB-200. With the minimal thermal expansion exhibited by UoB-200 (see above), our DFT simulated ambient pressure volumes similarly represent a good description for the ambient pressure geometries obtained within the diamond anvil cell (at ambient temperature).

The pressure-volume curves obtained for UoB-200 using PBE-D3 and PBE-D4 are effectively identical, with a slightly larger volume calculated at PBE-D3 level across the entire pressure range (Fig. 3c). Both simulated curves sit at lower volume as compared with the experimental data points. However, even though our DFT simulations are performed on de-solvated UoB-200 structure, we observe an extremely good comparison between the experimental and simulated compressibility over the experimentally accessible pressure range (<3.5 GPa). In fact, when the relative compressibility is considered, there is effectively no difference observed between the simulated and experimental data sets. This strongly suggests that, at low pressure, the solvent has negligible influence on the mechanical stability of UoB-200, whilst also lending support to the validity of our simulations to study the mechanical response of UoB-200 beyond experimentally accessible pressures.

A fit of the simulated pressure-volume curve of UoB-200 to the 2nd and 3rd order Birch–Murnaghan equation, yields a bulk modulus of ∼15.5 GPa (2nd order) and ∼13.4 GPa (3rd order), Table 1. Owing to the limited data points, the fits to the experimental data yield slightly lower and statistically less reliable bulk moduli of 13.952 GPa (2nd order) and 9.677 GPa (3rd order). Our obtained values of bulk modulus are consistent with values that are typical of framework materials.^11,37^

**Table 1:** Bulk moduli and derivatives for 2nd and 3rd order BM equation of state

	2nd order		3rd order	
	*B*_0_ GPa	*B*′	*B*_0_ GPa	*B*′
Experiment	13.95 ± 1.05	—	9.68 ± 1.56	8.40 ± 1.92
D3	15.55 ± 0.38	—	12.99 ± 0.97	4.74 ± 0.29
D4	15.93 ± 0.41	—	13.37 ± 1.09	4.74 ± 0.33

Upon compression, all three crystallographic axes compress monotonically (see SI S4), with positive compressibility observed in the *a* = *b* crystallographic directions, and negative linear compressibility in the crystallographic *c* direction. This is consistent with the experimental observation of an increased *W* : *H* ratio for the pores upon compression, and is consistent with the behaviour observed when wine-rack MOF topologies are compressed.^36^ From the simulated compressibility curves, we obtain values for the linear compressibility up to 10 GPa of *K*_LC_^*a*=*b*^ = 20.32 TPa^−1^ and *K*_LC_^*c*^ = −11.14 TPa^−1^ at DFT-D3 level, and *K*_LC_^*a*=*b*^ = 19.51 TPa^−1^ and *K*_LC_^*c*^ = −9.25 TPa^−1^ at DFT-D4 level. At low pressures (*i.e.* up to 2 GPa) these values are significantly larger, with *K*_LC_^*a*=*b*^ = 42.99 TPa^−1^ and *K*_LC_^*c*^ = −28.90 TPa^−1^ for DFT-D3 simulations and *K*_LC_^*a*=*b*^ = 39.23 TPa^−1^ and *K*_LC_^*c*^ = −21.08 TPa^−1^ for DFT-D4 simulations. While we do not have enough experimental data points to compare the NLC behaviour to 2 GPa, a calculation to 3.4 GPa gives values of *K*_LC_^*a*=*b*^ = 32.83 TPa^−1^ and *K*_LC_^*c*^ = −18.36 TPa^−1^, as compared with *K*_LC_^*a*=*b*^ ≈ 34 TPa^−1^ and *K*_LC_^*c*^ ≈ −20 TPa^−1^ averaged from our DFT models at to the same pressure range (see SI S4), suggesting our simulated values predict comparable mechanical behaviour, despite not including solvent molecules in the MOF pores. This is consistent with the alignment of our DFT simulated pressure-volume curves in Fig. 3.

Until 2013, the record non-linear compressibility over similar pressure ranges was held for KMn[Ag(CN)_2_]_3_, with *K*_NLC_ = −12.0 TPa^−1^. This record was beaten by the material Zn[Au(CN)_2_]_2_ which had, over a similar pressure range (0–1.8 GPa) *K*_LC_ = −45 TPa^−1^, though such large NLC effects remain extremely rare over these pressure windows.^29^ We note that while larger values of NLC are known (*e.g. K*_LC_ = −62.4 TPa^−1^ in InH(BDC)_2_), these values are obtained over much smaller pressure ranges (<1 GPa) and are therefore not directly comparable with our material. Whilst not a record breaker, the magnitude of NLC reported in our material, particularly over this large pressure range, is uncommon, with only a small number of examples exhibiting similar NLC behaviour.^30^ The majority of framework materials that have been studied by high pressure techniques either exhibit all positive linear compressibility, or have NLC effects that are significantly smaller^59^ or only occur over much smaller pressure ranges.^31,60^

As the wine rack pores are compressed, our experimental data, see above, indicated a reduction in the available void volume. This behaviour is reproduced in our DFT simulations (Fig. 3d) which suggest that empty voids in UoB-200 could be expected to reduce in volume quasi-linearly at least up to 10 GPa. By this pressure the void volume in our simulated structures has decreased from 360 Å^3^ in the primitive unit cell to only 80 Å^3^. We note that this is smaller than the expected molecular volume for ethanol. In fact, given that the maximum number of ethanol molecules that could reside in our ambient pressure void volume is *ca* 3 whole molecules, we anticipate the solvent begins to play an important role in the mechanical stability of UoB-200 between 2–3 GPa, *i.e.* at void volumes where the void volume space is less than that required for 3 ethanol molecules. This is consistent with the pressure range at which the structural integrity of UoB-200 was lost and may indicate that much higher pressures could be achieved by compression of a de-solvated material.

**Fig. 5:**
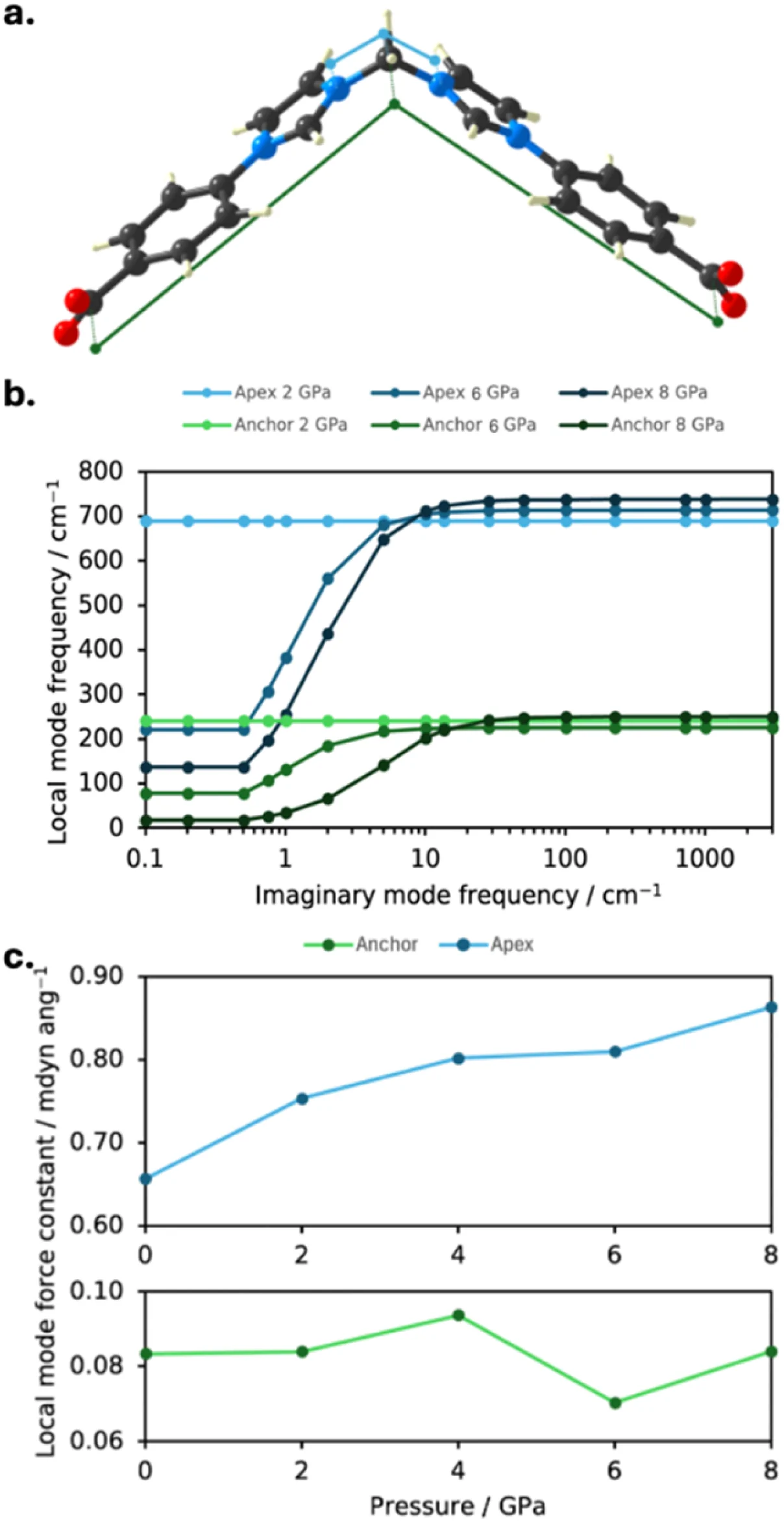
Local vibrational mode analysis of UoB-200 under variable pressure conditions. (a) The two internal coordinates used for local mode analysis, showing the ‘apex’ coordinate (blue) and the ‘anchor’ coordinate (green). (b) The effect that changing the imaginary mode frequency has on the calculated local mode frequencies for both internal coordinates at 2, 6 and 8 GPa. (c) The effect of pressure on the local mode force constant for both internal coordinates as a function of pressure.

We further assessed the effects of pressure on UoB-200, focusing on the flexibility of the linkers by performing local vibrational mode analysis (LMA) based on Brillouin zone-centre phonon frequencies (see SI S4)^61^ with extracted local mode frequencies and local mode force constants being indicative of the linker flexibility. With a focus on the effect of pressure on the UoB-200 linker flexibility, we defined two internal coordinates to define local modes for our analysis, Fig. 5a. The first coordinate was the N–C–N angle at the apex of the linker, which reflects the rigid bending of the linker molecule. The second coordinate was defined as the angle formed between the anchoring carboxylate carbons and the apex carbon, aiming to capture the broader bending motion of the full linker chain.

Our phonon calculations indicated that the removal of solvent molecules from the UoB-200 framework caused dynamical instabilities (imaginary frequencies) in some low-pressure structures that could not be converged out (see frequencies in SI S4.5). Notably, these instabilities were minor at low pressures, but increased significantly at 6 GPa, suggesting the solvent-free UoB-200 structure becomes increasingly dynamically unstable at higher pressures.

We first investigated the effect that these imaginary frequencies had on our LMA study, Fig. 5b, by systematically varying where in the phonon spectrum these imaginary frequencies might reside in a dynamically stable structure. Fortunately, the imaginary mode in the 2 GPa structure had no impact on the calculated local mode properties, as seen by the invariance of the local mode frequencies to the choice of its phonon frequency. In contrast, the instabilities in the 6 and 8 GPa structures cause the local mode properties to increase from softer to higher values, as the phonon frequency is increased. However, we note that once a phonon frequency of ∼30 cm^−1^ is reached, further increase has no effect on the local mode properties. Noting that the local mode frequencies and force constants for both our selected internal coordinates increase systematically across the stable 0, 2, and 4 GPa geometries, we posit that the 6 and 8 GPa behaviours are likely to keep with this trend and therefore performed our analysis to reflect the behaviour that our local modes would have if the imaginary phonon frequencies were >30 cm^−1^ for these structures.

With these considerations in mind, our LMA analysis reveals a general trend of stiffening local force constants, and hence a reduced flexibility of the MOF linker, as the pressure is increased, Fig. 5c. This is particularly evident for the bending mode at the apex of the linker, which stiffens by *ca* 22% when the pressure reaches 4 GPa, and by *ca* 31% at 8 GPa compared to ambient pressure. The force constants for the angle bending between the ‘anchor’ atoms of the linker also increase up to a pressure of 4 GPa, but for the dynamically unstable structures at 6 and 8 GPa the force constants soften. Whilst more detailed studies would be required, this may indicate that the dynamical instability in UoB-200 at higher pressures is associated with a delocalised deformation across the entire linker.

## Conclusions

UoB-200 exhibits both a highly unusual, entangled topology of 1D MOFs and large NLC effects over a significant pressure range. Such behaviour is unusual for all materials, not just MOFs, and is the first example of such NLC effects for a 1D MOF. Thus, it is apparent from our study that 1D MOFs are not only capable of exhibiting unusual NLC behaviour but that they offer properties that are competitive with 3D systems. The precise underpinning explanation for the performance of UoB-200 is interesting and may be due to a number of factors, including the 1D → 1D interpenetration in this MOF. Attempts to prepare a non-interpenetrated analogue have been unsuccessful thus far, possibly due to a number of related structures formed by the same combination of Cu(ii) and L.^52^ Our studies continue to explore the complex structural landscape associated with UoB-200. The behaviour of UoB-200 leads us to conclude that further studies seeking to identify other 1D MOFs, for example by tuning metal SBU or ligand design, are highly likely to yield further unusual behaviour as well as unusual topologies.

## Author contributions

The synthesis of materials and VT crystallographic studies were conducted by SLG. AL collected and analysed high-pressure single crystal X-ray data. AALM and MM performed the calculations and modelling experiments. NRC, AALM and MRP provided supervision. The project was conceived by NRC. All authors discussed the results, contributed to and have given approval to the final version of the manuscript.

## Conflicts of interest

There are no conflicts to declare.

## Supplementary Material

SC-017-D6SC02574A-s001

SC-017-D6SC02574A-s002

## Data Availability

CCDC 2491679–2491689 (studies of UoB-200 at different temperatures) and 2473944–2473949 (high pressure studies of UoB-200) contain the supplementary crystallographic data for this paper.^62*a–q*^ Supplementary information (SI): synthesis and crystal growth, details of crystallographic measurements and computational modelling. The X-ray crystallographic coordinates for structures reported in this study have been deposited at the Cambridge Crystallographic Data Centre (CCDC), under deposition numbers 2491679–2491689 (studies of UoB-200 at different temperatures, 2473944–2473949 (high pressure studies of UoB-200). See DOI: https://doi.org/10.1039/d6sc02574a.
