# Supplementary material for: Negative linear compressibility of a one-dimensional interpenetrated metal–organic framework
Source: Chem Sci. 2026 Jul 8. Online ahead of print. doi: 10.1039/d6sc02574a (PMC13371436; doi:10.1039/d6sc02574a)
Supplement: SC-OLF-D6SC02574A-s001 [file SC-OLF-D6SC02574A-s001.pdf]

Supplementary Information (SI) for Chemical Science.
This journal is © The Royal Society of Chemistry 2026

*Supplementary Information for:*

***Negative Linear Compressibility of a One-dimensional Interpenetrated Metal-organic Framework***

Sarah L. Griffin, Alexandra Longcake, Mateusz Mojsak, Adam A. L. Michalchuk, Michael R. Probert and Neil R. Champness

S1 Synthesis Page 2

S2 Experimental Details Page 4

S3 Effects of Pressure on UoB-200 Page 6

S4 Computational Details Page 8

S5 References Page 15

## S1 Synthesis

### *S1.1 Characterization.*

NMR spectra were recorded on a Bruker AVANCE NEO 400 MHz spectrometer and referenced to residual solvent peaks, unless otherwise stated. Deuterated solvents were used as specified. Chemicals used were all purchased from either Sigma Aldrich, ThermoFisher, Alfa Aesar, or Fluorochem and were used without further purification.

### *S1.2 Synthesis of methyl 4-(1H-imidazol-1-yl)benzoate (1)*

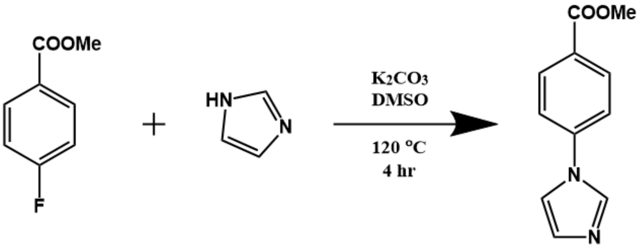


Methyl 4-fluorobenzoate (10 ml, 77.35 mmol), imidazole (10.5 g, 154.7 mmol), potassium carbonate (21 g, 152 mmol) and DMSO (30 ml) were combined in a round bottom flask. The mixture was heated at 120 °C for 4 hours. Upon completion the solution was cooled to room temperature before adding water. The product was extracted into ethyl acetate and washed with water (x2) and dried over magnesium sulphate. The resultant solution was rotary evaporated to dry, yielding a white solid. If impurities were present a diethyl ether recrystallisation was used to further purify the compound (13.45 g, 66.5 mmol, 86% yield). $^1$H NMR ($CDCl_3$): 3.94 (s, 3H), 7.24 (t, 1H), 7.34 (t, 1H), 7.47 (dt, 2H), 7.94 (t, 1H), 8.16 (dt, 2H); $^{13}$C NMR: 52.40, 117.76, 120.62, 129.03, 131.13, 131.57, 135.42, 140.73, 166.01.

### *S1.2 Synthesis of 3,3'-methylenebis(1-(4-(methoxycarbonyl)phenyl)-1H-imidazol-3-ium) (2)*

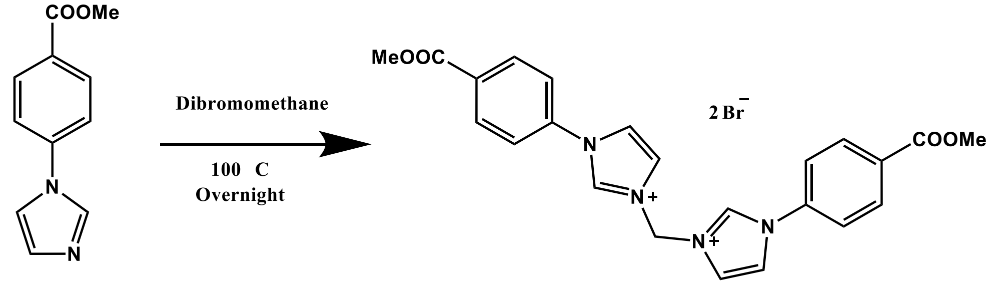


Compound **1** (1 g, 4.9 mmol) and dibromomethane (4 ml, 57 mmol) were combined in a round bottom flask. The mixture was heated at 100 °C for 24 hrs. Upon completion, the reaction was allowed to cool before removing excess dibromomethane via rotary evaporation. The resultant white solid was rinsed with dichloromethane before drying (1.2 g, 2.3 mmol, yield 92%). $^1$H NMR (DMSO-$d_6$): 3.92 (s, 6H), 6.69 (s, 2H), 8.02 (d, 4H), 8.26 (d, 4H), 8.43 (t, 2H), 8.55 (t, 2H), 10.45 (t, 2H); $^{13}$C NMR: 53.13, 59.14, 121.97, 122.76, 123.75, 131.42, 131.67, 138.29, 138.36, 165.57.

### *S1.3 Synthesis of 3,3'-methylenebis(1-(4-carboxyphenyl)-1H-imidazol-3-ium) ((L)$Cl_2$)*

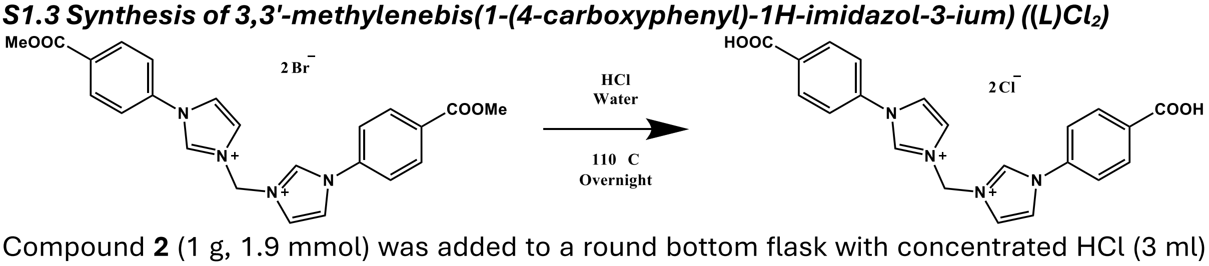


Compound **2** (1 g, 1.9 mmol) was added to a round bottom flask with concentrated HCl (3 ml) and water (12 ml). The mixture was heated at 110 °C overnight. Upon completion the solution was cooled to room temperature and the white precipitate filtered, washing with a minimal

amount of water due to solubility. The white solid, (**L**)$Cl_2$ was isolated and dried (0.78 g, 1.7 mmol, 89%). $^{1}H$ NMR (DMSO-$d_6$): 7.01 (s, 2H), 7.99 (d, 4H), 8.21 (d, 4H), 8.52 (t, 2H), 8.59 (t, 2H), 10.86 (t, 2H), 13.47 (s); $^{13}C$ NMR: 58.9, 121.81, 121.51, 123.82, 131.76, 132.70, 138.02, 138.48, 166.56.

***S1.4 Synthesis of UoB-200***

Linker **L** (10 mg, 0.022 mmol) was dissolved in water (5 ml) and neutralised with 0.1 M NaOH aq solution, being very careful not to exceed pH 7. Copper(II) nitrate hydrate (25 mg, 0.11 mmol) was dissolved in ethanol (10 ml). The ethanol metal solution was layered onto the aqueous linker solution, and the vial loosely lidded. The solution was left to crystallise for 1 week, after which a mix of green cube and blue plate crystals appeared. The green cube crystals were selected for single crystal XRD.

Crystal data UoB-200: $C_{42}H_{32}Cl_2Cu_2N_8O_8 \cdot Cl \cdot 7.5[H_2O]$, Mr = 1010.18, crystal dimensions 0.26 × 0.15 × 0.12 mm, Tetragonal, $a = b$ = 16.5416 (2) Å, $c$ = 17.4852 (5) Å, α = β = γ = 90 °, $V$ = 4784.38 (13) Å$^3$ , $T$ = 100 K, space group $I4/m$, $Z$ = 4, 16454 measured reflections, 2451 unique ($R_{int}$ = 0.037), which were used in all calculations. The final $R_1$ = 0.057 for 2303 observed data $R[F^2 > 2\sigma(F^2)]$ and $wR(F^2)$ = 0.166. Crystal structure data are available from the CCDC, deposition numbers CCDC 2491679-2491689 (studies at different temperatures), CCDC 2473944-2473949 (high pressure studies).

## S2. Experimental Details

### *S2.1 Variable temperature single crystal XRD*

Single crystal XRD data were collected using a Rigaku Synergy-S dual source with a PhotonJet-S X-ray source and a HyPix-6000 detector. Data were collected at varied temperatures through the use of an Oxford Cryosystems cryostream device. A single crystal was mounted on a mitogen loop using a minimum amount of fomblin oil to not impede solvent loss upon heating of the crystal. A full data collection was complete at 100K before heating to 400K and holding for 6 hrs to ensure full desolvation. The sample was then cooled to 100K, with complete data sets being collected at 100K, 150K, 200K, 250K, 300K, 350K, 400K, 300K, 200K and 100K.

**Table S2.1.** Unit cell data for UoB-200 obtained at variable temperatures from single crystal XRD measurements. Note, after de-solvation, the crystal was first heated from 100-300 K, then cooled to 100 K to verify recovery.

| | Temp. /K | *a* axis /Å | *c* axis /Å | Volume /Å$^3$ | Primitive Cell Volume /Å$^3$ |
|---|---|---|---|---|---|
| | 100 solvated | 16.5416(2) | 17.4852(4) | 4784.38(13) | 2392.19 |
| Heating | 100 | 16.1346(2) | 17.8644(4) | 4650.56(16) | 2325.28 |
| Heating | 150 | 16.1772(2) | 17.8278(5) | 4665.57(17) | 2332.79 |
| Heating | 200 | 16.2268(2) | 17.7903(4) | 4684.35(16) | 2342.18 |
| Heating | 250 | 16.2725(2) | 17.7571(4) | 4701.98(16) | 2350.99 |
| Heating | 300 | 16.3153(2) | 17.7313(4) | 4719.88(16) | 2359.94 |
| Heating | 350 | 16.3728(2) | 17.6894(4) | 4741.97(16) | 2370.99 |
| Heating | 400 | 16.4259(3) | 17.6424(7) | 4760.1(3) | 2380.05 |
| Cooling | 300 | 16.3220(2) | 17.727(4) | 4722.61(16) | 2361.31 |
| Cooling | 200 | 16.2264(2) | 17.7878(4) | 4683.46(16) | 2341.73 |
| Cooling | 100 | 16.1326(2) | 17.8646(4) | 4649.45 | 2324.73 |

### *S2.2 Variable pressure single crystal XRD*

High pressure data for UoB-200 were collected at 293(2) K on a Rigaku XtaLAB Synergy-S diffractometer equipped with an Oxford Cryosystems Cryostream open-flow cooling device using mirror monochromated Mo Kα radiation (λ = 0.71073 Å) generated using a microfocus sealed X-ray tube source and detected at a HyPix Arc-100 Detector. A crystal of UoB-200 (green block) was cut to dimensions of approximately 0.06 × 0.06 × 0.20 mm and studied in Daphne-7575 at pressures of 0.0, 1.37(5), 2.14(5), 2.87(5), 3.44(5) and 4.04(5) GPa, with viable datasets suitable for unit cell indexing and structural analysis obtained up to 3.44(5) GPa. At the final pressure point of 4.04(5) GPa, sample amorphisation was observed, so this datapoint is not mentioned further in the discussion. The hydrostatic limit of Daphne-7575 is reported to be approximately 4 GPa,[S1,S2] and was expected to be a non-penetrating pressure transmitting medium for compression studies.

The sample chamber of the two screw Merrill-Bassett diamond anvil cell (DAC) used for high pressure studies was formed by two 800 μm culet faces of Boehler-Almax diamonds fitted into tungsten carbide backing seats. A stamped steel sheet (thickness 250 μm) was indented to a thickness of approximately 140 μm to form the gasket. The gasket hole was drilled using a 380 μm diameter electrode on a BETSA electric discharge machine. The sample crystal was fixed to one culet face by means of high vacuum hydrocarbon grease and two ruby spheres, which allowed for pressure measurement using the ruby fluorescence method.[S3] After each pressure ramp, the pressure inside the DAC was allowed to equilibrate for a minimum of 24 hours before data collection was initiated. Pressure measurements were taken immediately before and after each collection and the pressure reported as the average. Individual dataset error bars are supplied on figures as 0.5 times the magnitude of the individual pressure drift plus 0.05 GPa, attributed to the inherent uncertainty in the pressure determination given by the ruby fluorescence method.[S4] Error bars associated with bond lengths and angles for specific refinements are supplied on figures as the estimated standard deviation (esd) as calculated by SHELXL.[S5]

Cell refinement and data reduction were carried out using the software CrysAlisPRO.[S6] Special settings (DAC opening angle, dataset resolution limits, profile rejection parameters and regular background updates) were implemented in the data reduction step, which helped remove contaminating diamond reflections and powder rings from the data. Individual specifications of the exact settings used are contained within the CIFs in the field '_diffrn_special_details'. Multi-scan absorption corrections were applied with an empirical absorption correction using spherical harmonics, implemented in the SCALE3 ABSPACK scaling algorithm[S7] through CrysAlisPRO.[S6] The initial collection conducted outside of the DAC at ambient pressure was solved using SHELXT[S8] and all high pressure datasets conducted inside the DAC were solved by importing a reference model from either this ambient condition collection or an appropriate model from the previous lower pressure point. Structural refinements were carried out using SHELXL[S5] with the Olex2 interface.[S9] For all high pressure datasets, in order to preserve the data-to-parameter ratio as much as possible, disorder of the organic linker was not modelled, allowing for the refinement of the anisotropic displacement parameters for all non-hydrogen atoms. Specific refinement details are supplied within the individual CIFs in the field '_refine_special_details'.

**S3 Effect of Pressure on UoB-200**

**S3.1 Experimental high-pressure study of the UoB-200 unit cell geometry**

The unit cell parameters collected for UoB-200 were determined up to 3.44 GPa, across 5 pressure points, Figure S4.1. Smooth, monotonic compression of both the *a* and *b* axes were observed across the studied pressure range, alongside concomitant elongation of the c axis, Fig S4.1 and Table S4.1. Although further data points would be required in the low pressure regime to confirm the following observation, it appears that the structure compresses more readily within the first 1-1.5 GPa, compared to pressures above 1.5 GPa. This behaviour is expected, since void space is removed readily at modest pressures from porous systems, such as UoB-200. However, due to the lack of datapoints in this initial pressure region, this observation cannot be fully substantiated in a quantitative manner from this initial study. Between the pressures of 0 GPa and 3.44 GPa, the unit cell volume decreased by approximately 16% from 4813.5(6) Å$^3$ to 4026.9(4) Å$^3$.

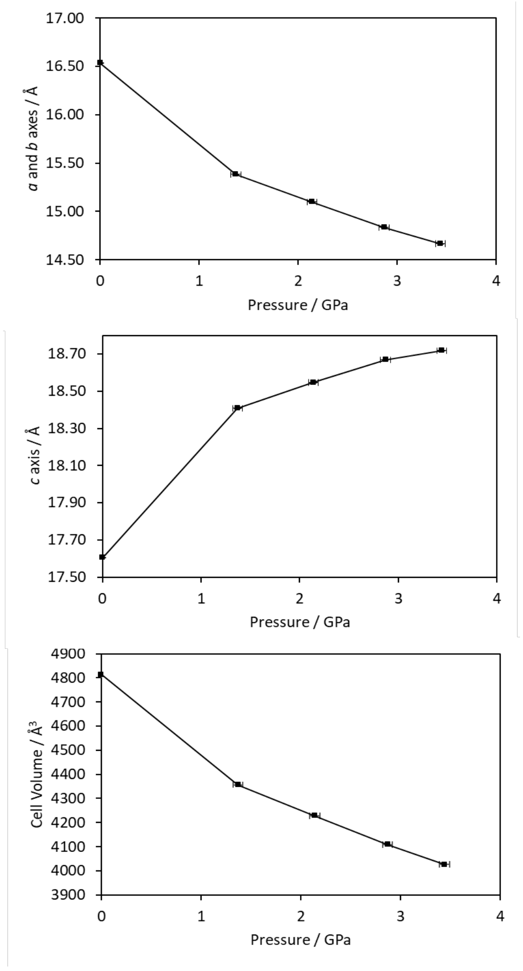


**Figure S3.1**. Unit cell parameters for UoB-200 as a function of pressure.

**Table S3.1** Experimental lattice constants obtained from high-pressure single crystal X-ray diffraction studies of UoB-200

| Pressure /GPa | a=b axis /Å | c axis /Å | Volume /Å$^3$ |
|---|---|---|---|
| 0 | 16.5352 | 17.6053 | 4813.52 |
| 1.37 | 15.3845 | 18.4083 | 4356.93 |
| 2.14 | 15.0993 | 18.5469 | 4228.49 |
| 2.87 | 14.8370 | 18.6703 | 4110.02 |
| 3.44 | 14.6677 | 18.7177 | 4026.95 |

The solvent accessible void space was calculated using the solvent mask based on the PLATON SQUEEZE[S10] routine implemented through Olex2, using a probe radius of 1.2 Å, Table S4.2. The converged models with no solvent modelled were fully constrained using the AFIX 1 command before a solvent mask was applied, allowing an estimation of the solvent contribution to the structure factors to be obtained. The solvent in UoB-200 could not be appropriately modelled due to extensive disorder and the limited completeness and resolution of the high-pressure datasets. The low angle reflections that deviated significantly from the $F_{obs}$-$F_{calc}$ graph were not omitted, since the low angle reflections are heavily influenced by disordered solvent. Additionally, strong reflections should not be omitted from refinements, if possible, especially for high pressure datasets where dataset resolution and completeness are already limited.

**Table S3.2.** Table of the solvent accessible void space and the number of electrons recovered for the datasets of UoB-(200) at various pressures, as calculated by the solvent mask procedure implemented in Olex2.

| Pressure /GPa | Total solvent accessible void volume /Å$^3$ | No. electrons recovered | Approx. No. EtOH molecules in pores |
|---|---|---|---|
| 0.0 | 991 | 257 | 10 |
| 1.37(5) | 712 | 250 | 10 |
| 2.14(5) | 651 | 272 | 10 |
| 2.87(5) | 592 | 271 | 10 |
| 3.44(5) | 544 | 266 | 10 |

## S4 Computational Details

All computational modelling was performed within the framework of plane wave density functional theory (DFT) as implemented in CASTEP v24.[S11] Input structures were taken from low temperature single crystal X-ray diffraction data, collected as described above. Modelling was performed with the Generalized Gradient Approximation functional of Perdew-Burke-Ernzerhof (PBE),[S12] alongside the semi-empirical dispersion corrections of Grimme (D3[S13] and D4[S14]). The ion cores were modelled using ultrasoft pseudopotentials generated on the fly as implemented in CASTEP. The wave function was expanded in plane waves to a kinetic energy cut-off of 900 eV and the electronic structure was sampled on a Monkhorst-Pack grid[S15] of spacing 0.07 $A^{-1}$. Convergence of the self-consistent field (SCF) calculations was accepted with electronic energies on SCF loops achieved < 1E-8 eV and eigenvalues of individual cycles achieved < 1E-10 eV. For the structural relaxation convergence was accepted with total energy convergence < 3E-6 eV and maximum residual forces of 5E-3 $eV.A^{-1}$. Indicatrix calculations were performed using the open-source Pascal tool[S16] and void volumes calculated using Mercury.[S17]

### S4.1 Computational high-pressure study of the UoB-200 unit cell geometry

Our high-pressure simulations were performed at both DFT-D3 and DFT-D4 levels of theory, and the corresponding unit cell dimensions are given in Table S4.1. Our simulated pressure-volume curves were fit to Birch-Murnaghan equations of state, Figure S4.1. We note a better fit to the simulated data of the 3rd order Birch-Murnaghan equation of state.

Due to the orthogonal axes used to define the conventional unit cell, the principal strain axes are parallel to the crystallographic axes, such that $a = b = \mathrm{X}_{1/2}$ and $c = \mathrm{X}_3$. From the simulated compressibility curves, we obtain values for the linear compressibility of direction $\mathrm{X}$,

$$\mathrm{K}_{lc,l} = -\frac{1}{X}\frac{dX}{dp}$$

This yields, across the 10 GPa pressure range, $\mathrm{K}_{lc,_{1/2}} = 20.32$ $\mathrm{TPa}^{-1}$ and $\mathrm{K}_{lc,_{3}} = -11.14$ $\mathrm{TPa}^{-1}$ at DFT-D3 level, and $\mathrm{K}_{lc,_{1/2}} = 19.51$ $\mathrm{TPa}^{-1}$ and $\mathrm{K}_{lc,_{3}} = -9.25$ $\mathrm{TPa}^{-1}$ at DFT-D4 level. At low pressures (*i.e.* up to 2 GPa) these values are significantly larger, with $\mathrm{K}_{lc,_{1/2}} = 42.99$ $\mathrm{TPa}^{-1}$ and $\mathrm{K}_{lc,_{3}} = -28.90$ $\mathrm{TPa}^{-1}$ for DFT-D3 simulations and $\mathrm{K}_{lc,_{1/2}} = 39.23$ $\mathrm{TPa}^{-1}$ and $\mathrm{K}_{lc,_{3}} = -21.08$ $\mathrm{TPa}^{-1}$ for DFT-D4 simulations. A full summary of linear compressibility is in Table S4.3.

**Table S4.1**. Unit cell parameters for UoB-200, calculated using DFT-D3 and DFT-D4 methodologies. Data are reported for the conventional (tetragonal) unit cell geometry. Values for length X are given in Å and those for $K_{lc}$ are given in $\mathrm{TPa}^{-1}$.

| Pressure /GPa | a=b axis | | | | c axis /Å | | | |
|---|---|---|---|---|---|---|---|---|
| | DFT-D3 | | DFT-D4 | | DFT-D3 | | DFT-D4 | |
| | $X$ | $K_{lc}$ | $X$ | $K_{lc}$ | $X$ | $K_{lc}$ | $X$ | $K_{lc}$ |
| **0** | 15.9362 | | 15.6898 | | 18.2484 | | 18.5689 | |
| **1** | 15.2693 | 41.8500 | 15.0362 | 41.6607 | 18.6090 | -19.7579 | 18.8894 | -17.2617 |
| **2** | 14.5662 | 42.9852 | 14.4587 | 39.2337 | 19.3035 | -28.9083 | 19.3520 | -21.0861 |
| **3** | 14.1443 | 37.4803 | 14.0455 | 34.9349 | 19.6089 | -24.8504 | 19.6378 | -19.1877 |
| **4** | 13.8538 | 32.681 | 13.7658 | 30.6574 | 19.7537 | -20.6216 | 19.7706 | -16.1791 |

| | | | | | | | | |
|---|---|---|---|---|---|---|---|---|
| **5** | 13.6238 | 29.0214 | 13.5421 | 27.3768 | 19.8503 | -17.5558 | 19.8650 | -13.9599 |
| **6** | 13.4259 | 26.2534 | 13.3521 | 24.8325 | 19.926 | -15.3208 | 19.9291 | -12.2089 |
| **7** | 13.2447 | 24.1277 | 13.1765 | 22.8838 | 19.9977 | -13.6943 | 19.9954 | -10.9746 |
| **8** | 13.0661 | 22.5122 | 13.0016 | 21.4169 | 20.0796 | -12.5433 | 20.0743 | -10.1342 |
| **9** | 12.8819 | 21.2954 | 12.8201 | 20.3228 | 20.1791 | -11.7557 | 20.1681 | -9.5694 |
| **10** | 12.6975 | 20.3228 | 12.6275 | 19.5180 | 20.2815 | -11.1410 | 20.2869 | -9.2523 |

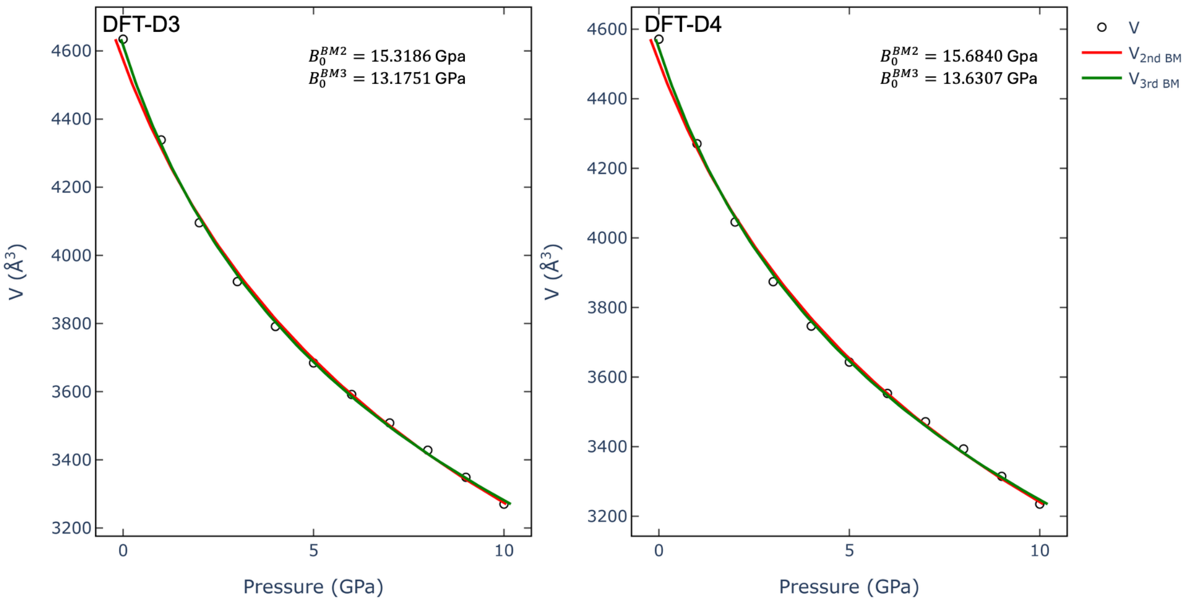


**Fig S4.1.** Fits of the Birch-Murnaghan equation of state (second order BM2 and third order BM3) to the computed pressure-volume data for UoB-200. The corresponding bulk moduli, $B_0$ are given.

## S4.2 Computational high-pressure study of the UoB-200 - local vibrational mode analysis

### Structural optimization

Initial geometries were taken from the ambient pressure experimental geometry obtained from single crystal X-ray diffraction. Calculations were performed using plane wave Density Functional Theory (DFT) as implemented in CASTEP v24[S18] using the generalized gradient approximation (GGA) functional of Perdew-Burke-Ernzerhof[S19] (PBE) and the semi-empirical dispersion correction of Grimme, D2.[S20] This dispersion correction was used to allow access to phonon calculations via the linear response method, as implemented in CASTEP v24. The nuclear-electron interactions were attenuated using norm-conserving pseudopotential as obtained on the fly in CASTEP, with the wavefunction approximated using a plane wave expansion to a maximum kinetic energy cut-off of 1000 eV. The electronic structure was sampled on a 2 x 2 x 2 Monkhorst-Pack grid.[S15] Electronic convergence accepted once the electronic energy reached < 1E-10 eV/atom, the electronic eigenvalues attained < 1E-12 eV/atom, and the

electronic force contribution was <1E-7 eV/Å. The geometries were considered converged once residual atomic forces < 2E-4 eV/Å. Phonon calculations were performed using the linear response implementation in CASTEP v24,[S21] using a fine grid scale for the real space charge density representation of 4.0.[S22] Local vibrational mode analysis was performed using a custom-built software (LModeA-k),[S23] using the dynamical matrices obtained from PBE-D2 calculations.

**Table S4.2**. Comparison of PBE-D2 geometry, optimized at 0 GPa, compared with the low temperature desolvated (100 K) experimental geometry.

| | **a=b / Å** | **c / Å** | **Vol / Å$^3$** |
|---|---|---|---|
| **Experiment** | 16.1326 | 17.8646 | 4649.45 |
| **PBE-D2** | 15.7562 | 18.4751 | 4586.56 |
| Δ /% | -2.33 | 3.30 | -1.35 |

**DFT-D3 / D4 FULL HIGH PRESSURE DATA SET**

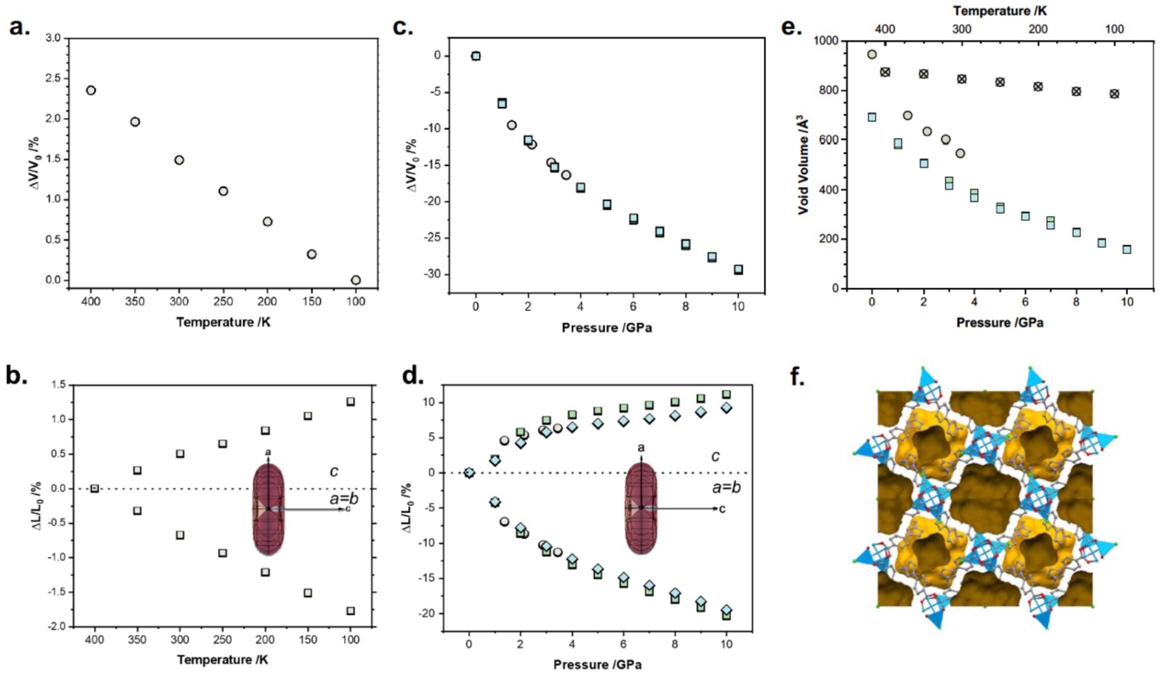


**Figure S4.2.** The effect of stress on the structure of the conventional unit cell of UoB-200. (a) The effect of temperature on the unit cell volume; and (b) on the crystallographic axes. The inset shows the thermal expansion indicatrix[50] along the crystallographic *b* axis. (c) The effect of hydrostatic pressure on the unit cell volume; and (d) on the unit cell axes. The inset shows the compressibility indicatrix along the crystallographic *b* axis. (e) The effects of temperature and pressure on the void volume, indicated as yellow in the associated crystal structure diagram (f). Experimental variable temperature data are shown as grey crossed circles.

**DFT-D2 HIGH-PRESSURE BEHAVIOUR.**

As the MOF was compressed hydrostatically, we observed monotonic compression, Table S4.3. A fit to the Birch-Murnaghan 2nd and 3rd order equations of state yield a bulk moduli on par with the DFT-D3 and D4 datasets, with the 3rd order equation of state yielding a somewhat better fit to experimental data as compared with DFT-D3 and DFT-D4, Table S4.4 and Figure S4.3. This is

consistent with experiment and the other computational models used here, validating our use of the DFT-D2 model for subsequent studies of the phonon-related properties. Similar to the other computational models, our PBE-D2 simulations indicate negative linear compressibility of the crystallographic c-axis, akin to the PBE-D3 and PBE-D4 calculations.

**Table S4.3.** The effect of hydrostatic compression on the unit cell response of MOF, calculated at PBE-D2 level of theory

| | **a=b / Å** | **c / Å** | **Vol / Å$^3$** |
|---|---|---|---|
| **0 GPa** | 15.7562 | 18.4751 | 4586.5555 |
| **1 GPa** | 14.8293 | 19.2219 | 4227.0582 |
| **2 GPa** | 14.3669 | 19.4999 | 4024.9480 |
| **3 GPa** | 14.0537 | 19.6567 | 3882.3110 |
| **4 GPa** | 13.8167 | 19.7461 | 3769.5750 |
| **5 GPa** | 13.5673 | 19.8151 | 3647.3980 |
| **6 GPa** | 13.4070 | 19.9159 | 3579.8241 |
| **7 GPa** | 13.1879 | 19.9813 | 3475.1714 |
| **8 GPa** | 13.0147 | 20.1320 | 3410.0186 |

**Table S4.4**. The bulk modulus and its derivative, obtained from fits to the Birch-Murnaghan equation of state, as implemented in the PASCal v2.2.0 code.[8] Data are given for the experimental data and PBE-D3/D4 data (see main manuscript for discussion), alongside the PBE-D2 data.

| | 2$^{nd}$ Order | | 3$^{rd}$ Order | |
|---|---|---|---|---|
| | $B_0$ GPa | $B'$ | $B_0$ GPa | $B'$ |
| Experiment | 13.952 $\pm$1.049 | - | 9.678 $\pm$ 1.565 | 8.402 $\pm$ 1.917 |
| D2 | 16.561 $\pm$ 0.846 | - | 11.677 $\pm$ 1.628 | 6.072 $\pm$ 0.799 |
| D3 | 15.319 $\pm$ 0.454 | - | 13.175 $\pm$ 1.161 | 4.636 $\pm$ 0.350 |
| D4 | 15.684 $\pm$ 0.486 | - | 13.631 $\pm$ 1.297 | 4.603 $\pm$ 0.385 |

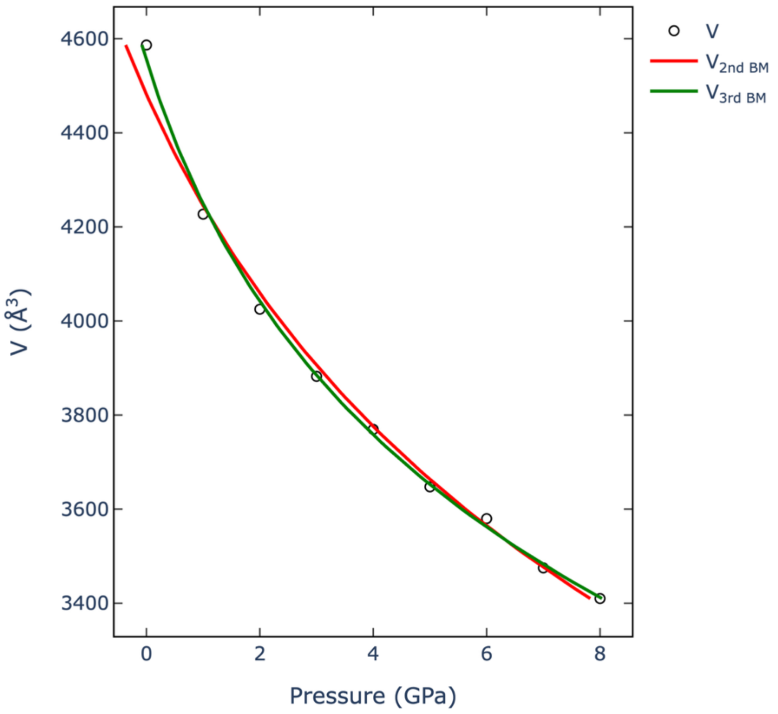


**Fig S4.3** The pressure-volume curve for UoB-200, calculated with the PBE-D2 level of theory. The data (circles) are fit to the 2nd (red) and 3rd (green) order Birch-Murnaghan equation of state.

**SET OF PHONON DENSITY OF STATES PLOTS**

The phonon frequencies for UoB-200 at a series of pressures was calculated at the PBE-D2 level of theory, Figure S4.4. Despite our best efforts, residual imaginary frequencies remained in number of the simulations, Table S4.5. The increase in imaginary modes with pressure suggests UoB-200 may become dynamically unstable at higher pressures.

**Table S4.5** List of imaginary frequencies in the simulated UoB-200 structure at each sampled pressure point.

| Mode | 0 GPa | 2 GPa | 4 GPa | 6 GPa | 8 GPa |
|---|---|---|---|---|---|
| **1** | -- | -54.91580 | -- | -28.115472 | -52.564052 |
| **2** | -- | -- | -- | -13.524777 | -51.467061 |
| **3** | -- | -- | -- | -- | -48.094055 |
| **4** | -- | -- | -- | -- | -41.547366 |
| **5** | -- | -- | -- | -- | -41.547366 |
| **6** | -- | -- | -- | -- | -13.560026 |
| **7** | -- | -- | -- | -- | -13.560026 |

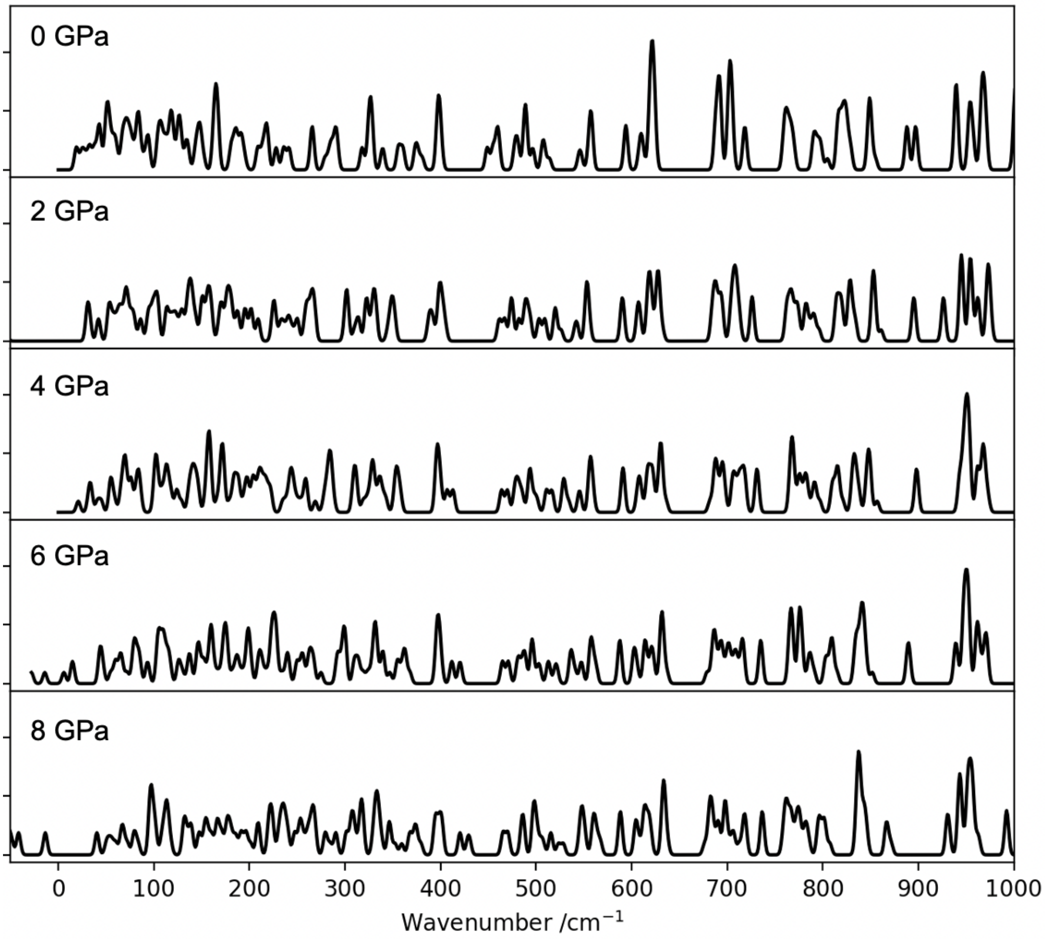


**Figure S4.4**. The phonon density of states calculated for UoB-200 at elevated pressures at the PBE-D2 level of theory. The density of states is shown only up to 1000 $cm^{-1}$, allowing the visualization of imaginary modes at higher pressures.

**EFFECT OF IMAGINARY MODES ON THE LOCAL MODE QUANTITIES**

Our tests (see main text) indicate that the local mode properties are best analysed by setting the imaginary phonon frequencies to large (positive) values. In the local mode formalism, this has the same effect as removing the eigenvectors associated with the imaginary frequencies from our local mode analysis altogether. Our analysis therefore assumes that the eigenvectors associated with the imaginary frequencies do not contribute to the local modes being analysed.

For practical purposes, force constants presented in the main text were computed by enforcing strictly zero contributions from the negative normal mode eigenvalues, achieved by setting the corresponding D matrix elements to zero. For the investigation of the impact of the imaginary frequencies on the local modes, the D matrix was left unmodified, but the negative normal mode eigenvalues were modified to correspond to selected positive normal mode frequencies. Where multiple normal mode frequencies were imaginary, all were set to the same positive frequency. Hence, we have effectively investigated their collective impact on the local modes, though is possible that some individual imaginary frequencies could have different effects on the local modes than what this collective behaviour reflects.

The local mode frequencies, force constants, and effective force constants are given in Tables S4.6 for the 'apex' local mode and in Table S4.7 for the 'anchor' local mode. Note that, to ensure direct comparison between values, the effective force constants are plotted in the main manuscript. Definitions of these different force constants can be found in Ref.[1]

**Table S4.6.** Local mode analysis results for the 'apex' local mode. Local mode frequencies are given in cm$^{-1}$, the force constant ($\kappa_{angle}$) is given in mdyn.Å.rad$^{-2}$, and the effective force constants ($\kappa_{eff}$) are given in mdyn.Å$^{-1}$.

| Pressure | $\omega_{loc}$ | $\kappa_{angle}$ | $\kappa_{eff}$ |
|---|---|---|---|
| 0 GPa | 644.4457 | 1.3937041 | 0.6566295 |
| 2 GPa | 689.4114 | 1.5940134 | 0.7533439 |
| 4 GPa | 710.4275 | 1.6905609 | 0.8015103 |
| 6 GPa | 713.6791 | 1.7065489 | 0.809675 |
| 8 GPa | 737.4141 | 1.8277693 | 0.8632107 |

**Table S4.7**. Local mode analysis results for the 'apex' local mode. Local mode frequencies are given in cm$^{-1}$, the force constant ($\kappa_{angle}$) is given in mdyn.Å.rad$^{-2}$, and the effective force constants ($\kappa_{eff}$) are given in mdyn.Å$^{-1}$.

| Pressure | $\omega_{loc}$ | $\kappa_{angle}$ | $\kappa_{eff}$ |
|---|---|---|---|
| 0 GPa | 231.646 | 6.9055932 | 0.0833266 |
| 2 GPa | 240.5907 | 6.9005885 | 0.083872 |
| 4 GPa | 257.188 | 7.6353448 | 0.0936416 |
| 6 GPa | 225.117 | 5.6736655 | 0.0702477 |
| 8 GPa | 249.4839 | 6.715775 | 0.0839572 |

**S5 References:**


S1 K. Murata and S. Aoki, *Rev. High Press. Sci. Technol.*, 2016, **26**, 3–7.

S2 D. Staško, J. Prchal, M. Klicpera, S. Aoki and K. Murata, *High Press. Res.*, 2020, **40**, 525–536.

S3 J. D. Barnett, S. Block and G. J. Piermarini, *Rev. Sci. Instrum.*, 1973, **44**, 1–9.

S4 W. B. Holzapfel, *J. Appl. Phys.*, 2003, **93**, 1813–1818.

S5 G. M. Sheldrick, *Acta Cryst.*, 2008, **A64**, 112–122.

S6 CrysAlisPRO, Oxford Diffraction /Agilent Technologies UK Ltd., Yarnton, England.

S7 R. C. Clark and J. S. Reid, *Acta Cryst.*, 1995, **A51**, 887–897.

S8 G. M. Sheldrick, *Acta Cryst.*, 2015, **A71**, 3–8.

S9 O. V. Dolomanov, L. J. Bourhis, R. J. Gildea, J. A. K. Howard and H. Puschmann, *J. Appl. Cryst.*, 2009, **42**, 339–341.

S10 A. L. Spek, *Acta Cryst.*, 2015, **C71**, 9–18.

S11 S.J. Clark, M.D. Segall, C.J. Pickard, P.J. Hasnip, M.I.J. Probert, K. Refson and M.C. Payne, *Z. Kristallogr*. 2005, **220**, 567–570.

S12 J.P. Perdew, K. Burke and M. Ernzerhof, *Phys. Rev. Lett*. 1997, **78**, 1396.

S13 S. Grimme, J. Antony, S. Ehrlich and H. Krieg, *J. Chem. Phys.*, 2010, **132**, 154104.

S14 E. Caldeweyher, J-M. Mewes, S. Ehlert and S. Grimme, *Phys. Chem. Chem. Phys.*, 2020, **22**, 8499-8512.

S15 H.J. Monkhorst and J.D. Pack, *Phys. Rev. B*, 1976, **13**, 5188.

S16 M.J. Cliffe and A.L. Goodwin, *J. Appl. Cryst.*, 2012, **45**, 1321-1329.

S17 C.F. Macrae, I. Sovago, S.J. Cottrell, P.T.A. Galek, P. McCabe, E. Pidcock, M. Platings, G.P. Shields, J.S. Stevens, M. Towler and P.A. Wood, *J. Appl. Cryst.*, 2020, **53**, 226-235.

S18 S. J. Clark, M. D. Segall, C. J. Pickard, P. J. Hasnip, M. I. J. Probert, K. Refson and M. C. Payne, *Z. Für Krist. - Cryst. Mater.*, 2005, **220**, 567–570.

S19 J. P. Perdew, K. Burke and M. Ernzerhof, *Phys. Rev. Lett.*, 1996, **77**, 3865–3868.

S20 S. Grimme, *J. Comput. Chem.*, 2006, **27**, 1787–1799.

S21 K. Refson, P. R. Tulip and S. J. Clark, *Phys. Rev. B*, 2006, **73**, 155114.

S22 M. Mojsak, T. M. Palmer and A. A. L. Michalchuk, *CrystEngComm*, 2026, **28**, 547-558.

S23 M. Mojsak, F. Bodo, A. Erba, A. A. L. Michalchuk and E. Kraka, *J. Chem. Theory Comput.*, 2026, acs.jctc.6c00097.
